# Homocysteine modulates 5‐lipoxygenase expression level via DNA methylation

**DOI:** 10.1111/acel.12550

**Published:** 2016-11-29

**Authors:** Jian‐Guo Li, Carlos Barrero, Sapna Gupta, Warren D. Kruger, Salim Merali, Domenico Praticò

**Affiliations:** ^1^ Department of Pharmacology and Center for Translational MedicineLewis Katz School of MedicinePhiladelphiaPA19140USA; ^2^ Department of Pharmaceutical SciencesTemple University PhiladelphiaPhiladelphiaPA19140USA; ^3^ Cancer Biology Program Fox Chase Cancer CenterTemple University PhiladelphiaPhiladelphiaPA19140USA

**Keywords:** 5‐lipoxygenase, Alzheimer's disease, amyloid beta, homocysteine, methylation, *S*‐adenosylmethionine

## Abstract

Elevated levels of homocysteinemia (Hcy), a risk factor for late‐onset Alzheimer's disease (AD), have been associated with changes in cell methylation. Alzheimer's disease is characterized by an upregulation of the 5‐lipoxygenase (5LO), whose promoter is regulated by methylation. However, whether Hcy activates 5LO enzymatic pathway by influencing the methylation status of its promoter remains unknown. Brains from mice with high Hcy were assessed for the 5LO pathway and neuronal cells exposed to Hcy implemented to study the mechanism(s) regulating 5LO expression levels and the effect on amyloid β formation. Diet‐ and genetically induced high Hcy resulted in 5LO protein and mRNA upregulation, which was associated with a significant increase of the *S*‐adenosylhomocysteine (SAH)/*S*‐adenosylmethionine ratio, and reduced DNA methyltrasferases and hypomethylation of 5‐lipoxygenase DNA. *In vitro* studies confirmed these results and demonstrated that the mechanism involved in the Hcy‐dependent 5LO activation and amyloid β formation is DNA hypomethylation secondary to the elevated levels of SAH. Taken together these findings represent the first demonstration that Hcy directly influences 5LO expression levels and establish a previously unknown cross talk between these two pathways, which is highly relevant for AD pathogenesis. The discovery of such a novel link not only provides new mechanistic insights in the neurobiology of Hcy, but most importantly new therapeutic opportunities for the individuals bearing this risk factor for the disease.

## Introduction

Alzheimer's disease (AD) is the most common form of chronic neurodegenerative condition with dementia in the elderly, affecting approximately 6–8% all persons aged > 65. While only a minority of AD cases is secondary to missense mutations in genes involved for either Aβ precursor protein (APP) or presenilin‐1 and presenilin‐2, the cause of sporadic AD remains unclear, and a combination of environmental and genetic risk factors has been implicated (Giannopoulos & Praticò, 2015). Considering that an effective treatment for AD is still unavailable, interventions aimed at controlling these risk factors can in the long run have a significant impact in reducing the number of cases and associated cost (Beydoun *et al*., 2014; Alzheimer's Association, 2015). Elevated levels of total plasma homocysteine (Hcy), also termed hyperhomocysteinemia (HHcy), are considered one modifiable risk factor for developing AD; however, the underlying mechanisms remain unknown (Zhuo *et al*., 2011; Shen & Ji, 2015).

Homocysteine is an intermediate metabolite of the methionine cycle, produced from the hydrolysis of *S*‐adenosylhomocysteine (SAH), which is a by‐product of methylation reactions involving the methyl donor *S*‐adenosylmethionine (SAM). As result, HHcy typically associates with elevation of intracellular SAH, a potent inhibitor of methyl‐transfer reactions, low SAM and ultimately DNA hypomethylation (Selhub, 1999).

The 5‐lipoxygenase (5LO) is an enzyme widely expressed in the central nervous system where its levels increase in an age‐dependent manner particularly in cortex and hippocampus, two brain regions highly vulnerable to neurodegenerative insults (Chinnici *et al*., 2007).

Previous works form our and other groups have shown that 5LO is upregulated in AD brains, and its modulation influences the development of the AD‐like phenotype of different transgenic mouse models of the disease (Chu *et al*., 2012a, 2013; Giannopoulos *et al*., 2014).

In addition to nucleotide polymorphism in the promoter region of the 5LO gene, other factors could influence its expression and ultimately drive its functional role in AD pathogenesis (Qu *et al*., 2001). Among them, epigenetic modifications have been implicated as important regulatory mechanism for this enzyme. In particular, 5LO expression seems to be tightly regulated by DNA methylation and de‐methylation of its promoter is a prerequisite for 5LO gene expression, protein synthesis and activity (Uhl *et al*., 2002).

Considering the importance of both genetic and environmental risk factors for late‐onset AD, one can envision a scenario in which 5LO (the gene) and HHcy (environmental factor) interact and together modulate disease pathogenesis. However, although they have been independently associated with AD, a mechanistic link between them has not been explored. We hypothesize that in a condition of HHcy, the increased intracellular SAH results in cellular hypomethylation that modulates 5LO gene expression influencing the neuronal cells phenotype and ultimately neurodegeneration.

## Results

### Diet‐induced HHcy

**Figure 1:**
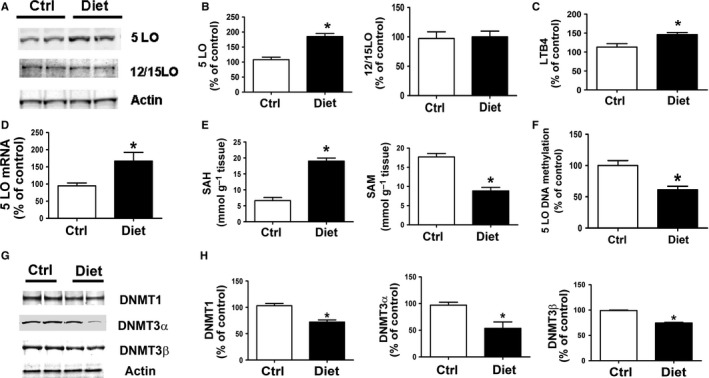
Diet‐induced HHcy upregulates brain 5LO pathway via DNA hypomethylation. (A) Western blot analysis of 5LO and 12/15LO protein levels in brain cortex homogenates from 3xTg‐AD mice with diet‐induced HHcy or 3xTg control. (B) Densitometric analysis of the immunoreactivity is shown in the previous pane. (C) Levels of LTB4 were assayed by ELISA in brain cortex homogenates from 3xTg‐AD mice with diet‐induced HHcy (closed bars) or controls (open bars). (D) Quantitative real‐time reverse transcription–polymerase chain reaction (qRT–PCR) analysis of 5LO mRNA in brain cortices of 3xTg mice with diet‐induced HHcy (closed bars) or controls (open bars). (E) Levels of SAH and SAM were assayed by HPLC in brain cortices of 3xTg‐AD mice with diet‐induced HHcy (closed bars) or controls (open bars). (F) 5LO DNA methylation state in brain cortices of 3xTg‐AD mice with diet‐induced HHcy (closed bars) or controls (open bars). (G) Western blot analyses for DNMT1, DNMT3α, and DNMT3β in brain cortices of 3xTg‐AD mice with diet‐induced HHcy (closed bars) or controls (open bars). (H) Densitometric analyses of the immuno‐reactivity are shown in the previous panel. Data presented are mean ± SEM (*P < 0.05, *n* = 6). HHcy, hyperhomocysteinemia; 5LO, 5‐lipoxygenase.

To start investigating the role that Hcy has on the expression levels of the 5LO enzymatic pathway, we examined brain cortices from 3xTg‐AD mice with diet‐induced HHcy (Li *et al*., 2014). Compared with controls, brain cortex homogenates from these mice had a significant increase in the steady state levels of 5LO protein, but no changes were observed for another lipoxygenase, 12/15LO (Fig. 1A,B). This increase was associated with a significant elevation of the leukotriene B4 (LTB4) levels, a 5LO major metabolic product, and 5LO mRNA levels (Fig. 1C,D). Consistent with the dietary HHcy condition, the same samples had higher levels of *S*‐adenosyl‐homocysteine (SAH) but lower *S*‐adenosyl‐methionine (SAM) (Fig. 1E). The altered SAH/SAM ratio resulted in a significant reduction in the methylation of the 5LO DNA, which was associated with a significant decrease in all three major DNA methyl‐transferase enzymes, DNMT1, DNMT3α, and DNMT3β (Fig. 1F–H).

### Genetic‐induced HHcy

**Figure 2:**
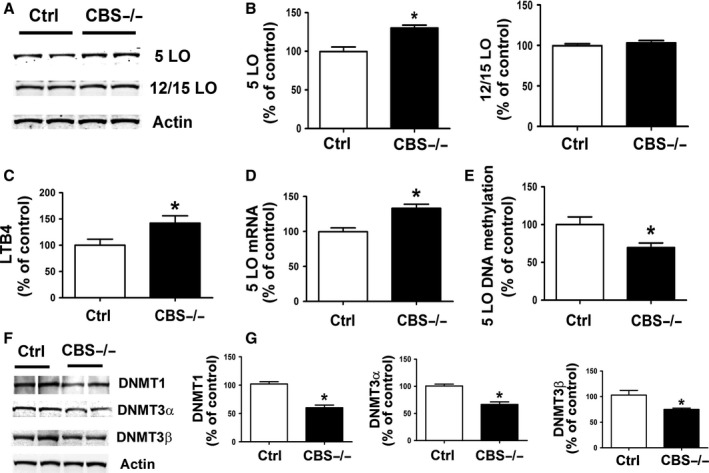
Genetically induced HHcy results in brain 5LO upregulation via DNA hypomethylation. (A) Western blot analysis of 5LO and 12/15LO in brain cortex homogenates from cbs−/− mice or control. (B) Densitometric analysis of the immunoreactivity is shown in the previous pane. (C) Levels of LTB4 were assayed by ELISA in brain cortex homogenates from cbs−/− mice (closed bars) or controls (open bars). (D) Quantitative real‐time reverse transcription–polymerase chain reaction (qRT–PCR) analysis of 5LO mRNA in brain cortices of cbs−/− mice (closed bars) or controls (open bars). (E) 5LO DNA methylation state in brain cortices of cbs−/− mice (closed bars) or controls (open bars). (F) Western blot analyses for DNMT1, DNMT3α, and DNMT3β in brain cortices of cbs−/− mice (closed bars) or controls (open bars). (G) Densitometric analyses of the immuno‐reactivity are shown in the previous panel. Data presented are mean ± SEM (*P < 0.05, *n* = 6). HHcy, hyperhomocysteinemia; 5LO, 5‐lipoxygenase.

Compared with wild‐type mice, brain cortices from cystathionine β‐synthase deficient mice (cbs−/−), which spontaneously develop HHcy (Sapna *et al*., 2009), had a significant increase in 5LO protein levels, which was associated with higher LTB4 and 5LO mRNA levels (Fig. 2A–D). By contrast, no changes were observed for the steady state protein levels of 12/15LO (Fig. 2A,B). Moreover, the same mice had significant reduction in 5LO DNA methylation levels, which was associated with a significant decrease in the levels of DNMT1, DNMT3α, and DNMT3β (Fig. 2E–G).

### *In vitro* studies

**Figure 3:**
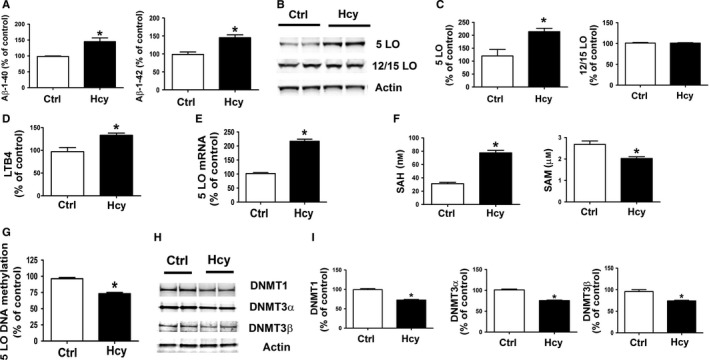
HHcy regulates 5LO expression levels and activity via its DNA methylation in neuronal cells. (A) Levels of Aβ1–40 and Aβ1–42 were assayed by ELISA in the supernatants of N2A‐APPswe cells incubated with vehicle (ctr) (open bars) or Hcy (50 μm) (closed bars). (B) Western blot analysis of 5LO and 12/15LO in lysates from neuronal cells incubated with Hcy or vehicle (ctr). (C) Densitometric analysis of the immunoreactivity is shown in the previous panel. (D) Levels of LTB4 were assayed by ELISA in supernatant from neuronal cells incubated with Hcy (closed bars) or vehicle (ctr) (open bars). (E) Quantitative real‐time reverse transcription–polymerase chain reaction (qRT‐PCR) analysis of 5LO mRNA in lysates from N2A‐APPswe cells incubated with Hcy (closed bars) or vehicle (ctr) (open bars). (F) Levels of SAH and SAM were assayed by HPLC in neuronal cells incubated with Hcy (closed bars) or vehicle (ctr) (open bars). (G) 5LO DNA methylation state in N2A‐APPswe cells incubated with Hcy (closed bars) or vehicle (ctr) (open bars). (H) Western blot analyses for DNMT1, DNMT3α, and DNMT3β in lysates from neuronal cells incubated with Hcy (closed bars) or vehicle (ctr) (open bars). (I) Densitometric analyses of the immuno‐reactivity are shown in the previous panel. Data presented are mean ± SEM (*P < 0.05, *n* = 3). HHcy, hyperhomocysteinemia; 5LO, 5‐lipoxygenase.

For these studies, we used the Neuro 2A (N2A) cells, a mouse neuroblastoma cell line stably expressing human APP carrying the K670 N, M671L Swedish mutation (N2A‐APPswe). To confirm the effect of Hcy on 5LO expression levels and its DNA methylation, we treated the N2A‐APPswe cells with 50 μm DL‐homocysteine for 24 h, and then, supernatant and cells lysates harvested for biochemistry analyses. As shown in Figure 3, we observed that compared with controls, cells exposed to Hcy had a significant increase in the amount of Aβ1–40 and Aβ1–42 levels (Fig. 3A) and 5LO protein levels, but no changes were detected for steady state levels of 12/15LO (Fig. 3B,C). These changes were associated with a significant increase in LTB4 as well as 5LO mRNA levels (Fig. 3D,E). In the same samples, we also observed a significant increase in cellular levels of SAH and a decrease in SAM (Fig. 3F), which was associated with a reduction in 5LO DNA methylation and steady state levels of DNMT1, DNMT3α, and DNMT3β (Fig. 3,G–I).

**Figure 4:**
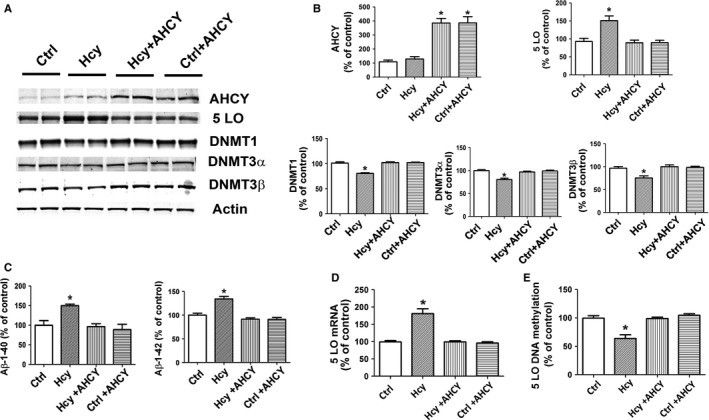
Hcy‐dependent 5LO DNA hypomethylation and activation requires intracellular SAH. (A) Western blot analysis of SAH hydrolase (AHCY), 5LO, DNMT1, DNMT3α, and DNMT3β in lysates from N2A‐APPswe cells incubated with vehicle (Ctrl), Hcy (50 μm), Hcy and transfected with AHCY cDNA, and vehicle and transfected with AHCY cDNA. (B) Densitometric analyses of the immunoreactivity are shown in the previous panel. (C) Levels of Aβ1–40 and Aβ1–42 were assayed by ELISA in the supernatants of the same cells shown in panel A. (D) Quantitative real‐time reverse transcription–polymerase chain reaction (qRT–PCR) analysis for 5LO mRNA in N2A‐APPswe cells incubated with vehicle (Ctrl), Hcy, Hcy and transfected with AHCY cDNA, and vehicle and transfected with AHCY cDNA. (E) 5LO DNA methylation in N2A‐APPswe cells incubated with vehicle (Ctrl), Hcy, Hcy and transfected with AHCY cDNA, and vehicle and transfected with AHCY cDNA. Data presented are mean ± SEM (*P < 0.05, *n* = 3). 5LO, 5‐lipoxygenase; SAH, *S*‐adenosylhomocysteine.

To prove a causative role for the elevation of S‐Adenosyl‐homocysteine Hydrolase (SAH) secondary to HHcy in the demethylation of 5LO DNA and upregulation of the 5LO pathway at the message and protein levels and Aβ formation, next we transfected the neuronal cells with SHA hydrolase (AHCY), which catalyzes the breakdown of SAH (Turner *et al*. 2000). Over‐expression of AHCY alone did not influence 5LO pathway or Aβ levels (Fig. 4A–C). By contrast, over‐expression of AHCY in the presence of Hcy prevented the upregulation of the 5LO pathway (protein and mRNA levels), the increase in Aβ formation (Fig. 4A–C), and the reduction in DNMT1, DNMT3α, DNMT3β (Fig. 4A,B), and 5LO DNA methylation (Fig. 4E).

**Figure 5:**
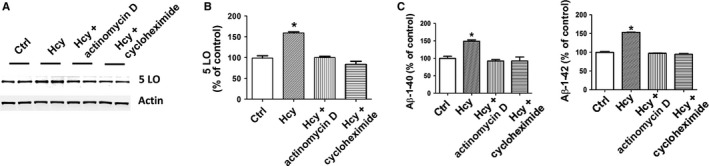
Hcy‐dependent 5LO upregulation requires mRNA transcription and protein synthesis. (A) Western blot analysis of 5LO in lysates from N2A‐APPswe cells treated with vehicle (Ctrl), Hcy (50 μm), Hcy plus actinomycin D (2 μg mL^−1^), and Hcy plus cycloheximide (1 μg ml^−1^). (B) Densitometry of the immunoreactivity is shown in the previous panel. (C) Levels of Aβ1–40 and Aβ1–42 were assayed by ELISA in supernatant from the same cells described in panel A. Data presented are mean ± SEM (*P < 0.05, *n* = 3). 5LO, 5‐lipoxygenase.

In another set of experiments, N2A‐APPswe cells exposed to high Hcy levels were also incubated with actinomycin‐D, inhibitor of mRNA translation, and cycloheximide, an inhibitor of protein synthesis, and the effect on 5LO and Aβ investigated. As shown in Figure 5, we found that compared with controls, Hcy induced an increase in 5LO protein and Aβ levels in the supernatant. However, these effects were blunted in the presence actinomycin‐D, suggesting that 5LO upregulation in the N2A‐APPswe cells results from an increase in transcription of 5LO mRNA (Fig. 5A–C). Cycloheximide also prevented the increase in 5LO protein and Aβ formation, suggesting that the 5LO upregulation is new protein synthesis‐dependent (Fig. 5A–C).

**Figure 6:**
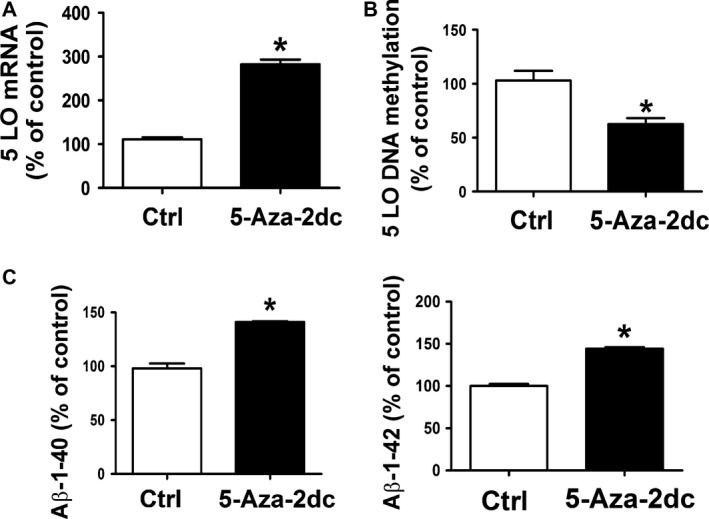
Effect of a DNA demethylating agent on 5LO mRNA and Aβ formation. (A) Quantitative real‐time reverse transcription–polymerase chain reaction (qRT–PCR) analysis of 5LO mRNA levels in N2A‐APPswe cells treated with 5‐aza‐2dc (5 μm) or vehicle (ctrl). (B) 5LO DNA methylation state in N2A‐APPswe cells incubated with 5‐Aza‐2dc (closed bars) or vehicle (ctr) (open bars). (C) Levels of Aβ1–40 and Aβ1–42 were assayed by ELISA in supernatant from the same cells described in the previous panel. Data presented are mean ± SEM (*P < 0.05, *n* = 3). 5LO, 5‐lipoxygenase.

Finally, to further demonstrate the susceptibility of 5LO mRNA transcription level to its DNA methylation state, N2A‐APPswe cells were incubated with 5‐Aza‐2dc, a potent de‐methylating agent. As shown in Figure 6, incubation with this agent by inducing a significantly reduction in 5LO DNA methylation resulted in a significant increase in its mRNA levels, which was associated with an elevation of Aβ1–40 and Aβ1–42 levels in the supernatant.

## Discussion

In the current article, we provide the first experimental evidence that high level of Hcy, also known as hyper‐Hcy or HHcy, by inducing an increase in intracellular SAH levels, and subsequent reduction in the methyltransferases activity results in 5LO DNA hypomethylation and ultimately upregulation of 5LO enzymatic pathway and Aβ formation.

Despite some conflicting data, most of the epidemiological and clinical studies have revealed that HHcy doubles the risk for developing AD independently of several other major factors (Ravaglia *et al*., 2003; Clarke *et al*., 2014). As HHcy occurs more frequently in old age, it has been proposed as a potential metabolic link for the frequent coexistence of aging and neurodegenerative diseases (Parnetti *et al*., 1997; Ravaglia *et al*., 2004) and several mechanisms have been proposed to explain this link. These include oxidative stress, excitotoxicity, cerebrovascular damage, and endoplasmic reticulum stress (Perna *et al*., 2003; Boldyrev & Johnson, 2007; Kim *et al*., 2008; Kuszczyk *et al*., 2009). However, the exact biochemical basis by which Hcy modulates neurodegenerative mechanisms relevant to AD remains unknown.

In search for a novel molecular mechanism linking HHcy with AD pathogenesis, in recent years, a methylation hypothesis has emerged in which Hcy elevation leads to a reduced ratio of SAM/SAH and decreased activity of methyltransferases which then may regulate gene expression (Caudill *et al*., 2001; Castro *et al*., 2005). In support of this hypothesis, our work shows that SAM levels are reduced and SAH levels increased both in the brains of mice with diet‐ and genetically induced HHcy and that this change is associated with an upregulation of the 5LO enzymatic pathway both at the protein and message levels. Interestingly, we found that HHcy has no influence on a distinct LO, the 12/15LO, suggesting a selectivity of its biological effect within the central nervous system. It is possible that the lack of effect for HHcy on the expression levels of the 12/15LO is secondary to the fact that, by contrast with the 5LO promoter, the former one is not affected by methylation changes in the central nervous system. Supporting this hypothesis, there are no data in the literature showing that the 12/15LO gene expression is modulated by epigenetic modifications in this particular system.

Previous work showed that folate deficiency, a condition associated with HHcy, increases the production of Aβ by affecting SAM‐dependent DNA methylation of genes involved in APP metabolism, such as PS1 (Fuso *et al*., 2005). Our finding add an extra facet to the neurobiology of HHcy and AD pathogenesis by showing that lowering SAM/SAH ratio influences the methylation of 5LO DNA, which results in its activation and increased Aβ formation. Considering the importance of both genetic and environmental risk factors in particular for late‐onset AD, our study implicates 5LO as the gene and HHcy as the environmental factor, which by interacting together modulates cellular events such as amyloidosis very relevant to the disease processes.

Because we showed that HHcy associates with SAH elevation, lower methyltrasferases, hypomethylation of 5LO DNA, its gene and protein upregulation, and increase in Aβ formation, next we wanted to prove a causative role for the elevation of SAH in these inter‐related biological effects.

To this end, we setup experiment with neuronal cells transiently over‐expressing SAH hydrolase, the only known enzyme to catalyze the breakdown of SAH and efficiently reducing its intracellular levels (Wang *et al*., 2014). These studies showed that SAH hydrolase over‐expressing cells were protected from the HHcy‐dependent 5LO upregulation and Aβ elevation whereby rescuing the HHcy‐dependent reduction in all 3 methyltransferase enzymes and 5LO hypomethylation. Additionally, we demonstrated that the effect of HHcy on the 5LO enzymatic pathway was secondary to an increase in transcription of 5LO mRNA rather than an increase in its stability as it was blocked by actinomycin‐D. Similarly, blockade of *de novo* protein synthesis by cycloheximide prevented the HHcy‐dependent upregulation of 5LO and increase in Aβ formation.

In summary, our studies reveal an unknown biological link between HHcy and 5LO enzyme and elucidate a novel pathway involving Hcy‐dependent SAH elevation, 5LO DNA hypomethylation, and Aβ formation. Their significance lies in the establishment of a mechanistic relationship between Hcy (environmental risk factor) and 5LO (genetic risk factor) with the pathogenesis of AD via an epigenetic mechanism. Taken together, they support the hypothesis that impaired Hcy metabolism and dysregulation of important methylation reactions can trigger the activation of an enzymatic pathway which ultimately favors amyloidogenesis and AD onset.

In conclusion, our findings provide novel insights into the molecular mechanisms by which elevated circulating Hcy levels may promote the development of AD‐like neuropathology in individuals carrying this environmental risk factor and ultimately afford us with useful information for novel therapeutic opportunities for them.

## Experimental procedures

### Mice and treatments

Animal procedures were approved by Temple University and Fox Chase Cancer Center Animal Care and Usage Committee and in accordance with the Guide for the Care and Use of Laboratory Animals of the National Institute of Health. The 3xTg‐AD mice harboring a human mutant APP (KM670/671NL), a human mutant PS1 (M146V) knock‐in, and tau (P301L) transgene (Oddo *et al*., 2003) used in this article were previously described (Li *et al*., 2014). Briefly, 3xTg‐AD mice were randomized into two groups: control group mice [*n* = 6 (three males and three females)] received standard rodent chow, whereas the Hcy‐diet group mice [*n* = 7 (two males and five females)] received a standard rodent chow deficient in folate (< 0.2 mg kg^−1^), vitamin B6 (< 0.1 mg kg^−1^), and B12 (< 0.001 mg kg^−1^), which is known to induce HHcy in mice, starting at 5 months of age for 7 months (Li *et al*., 2014).

### Mouse models of cystathionine β‐synthase deficiency (Tg‐I278T Cbs−/− mice)

The brain cortices of Tg‐I278T Cbs−/− mice and their control mice (Tg‐I278T Cbs +/+ and Tg‐I278T Cbs +/−) were also previously described (Sapna *et al*., 2009). These animals were about 3 months of age and all males. The mean serum total homocysteine for Tg‐I278T Cbs−/− mice was 296 μm compared to 5.5 μm for the controls (Sapna *et al*., 2009).

### Cell line and treatment

For our studies, we used the Neuro 2A (N2A) cells which derive from a mouse neuroblastoma cell line and have been extensively used to study neuronal differentiation, axonal growth, and signaling pathways. In particular, we used N2A cells stably expressing human APP carrying the K670 N, M671L Swedish mutation (N2A‐APPswe). Cells were cultured in Dulbecco's modified Eagle medium supplemented with 10% fetal bovine serum, 100 U mL^−1^ streptomycin (Cellgro, Herdon, VA), and 400 mg mL^−1^ G418 (Invitrogen, Carlsbad, CA, USA) at 37 °C in the presence of 5% CO_2_. They were cultured to 80% to 90% confluence in six‐well plates and then changed to fresh medium containing 50 μm DL‐homocysteine (Sigma, St Louis, MO, USA) with 40 μm adenosine (Sigma) and 10 μm erythro‐9‐(2‐hydroxy‐3‐nonyl)‐adenine hydrochloride (EHNA) (Sigma), as previously described (Zhuo *et al*., 2010; Li *et al*., 2014). After 24 h, media were collected for Aβ1–40 measurement and cell harvested for biochemistry analyses. For cell treatment, we use 5‐Aza‐2dc (Sigma) at 5 μm for 48 h, actinomycin D (Sigma) at 2 μg mL^−1^ for 18 h, or cycloheximide (Sigma) at 1 μg mL^−1^ for 13 h. For transfection studies, N2A‐APPswe cells were transfected with AHCY cDNA (Addgene, Cambridge, MA, USA) for 24–36 h using Lipofectamine 2000 (Invitrogen Corporation). Similarly to our previous publications using this very type of cells and the Lipofectamine 2000, under these experimental conditions, the transfection efficiency was always 75–80% (Chu *et al*., 2012a,b, 2015).

### Western blot analyses

RIPA fractions of brain cortex homogenates were used for Western blot analyses as previously described (Di Meco *et al*., 2014; Chu *et al*., 2015). Briefly, samples were electrophoresed on 10% Bis–Tris gels or 3–8% Tris–acetate gel (Bio‐Rad, Richmond, CA, USA), transferred onto nitrocellulose membranes (Bio‐Rad), and then incubated overnight with the appropriate primary antibodies. Antibodies against 5LO, 12/15LO, DNMT1, DNMT3α, and DNMT3β were obtained from Santa Cruz Biotech. (Dallas, TX, USA) and SAH hydrolase (AHCY) antibody from Novus Biologicals (Littleton, CO, USA). After three washings with T‐TBS, membranes were incubated with IRDye 800CW‐labeled secondary antibodies (LI‐COR Bioscience, Lincoln, NE, USA) at room temperature for 1 h. Signals were developed with Odyssey Infrared Imaging Systems (LI‐COR Bioscience). β‐Actin was used as internal loading control.

### Biochemical analyses

Levels of SAH and SAM were assayed as previously described (Skelly *et al*., 2003; Moncada *et al*., 2008). Briefly, tissues were frozen in liquid nitrogen and reduced into a fine powder using mortar and pestle. The powder was re‐suspended in 100 μL of NKPD buffer (2.68 mm KCl, 1.47 mm KH_2_PO_4_, 51.10 mm Na_2_HPO_4_, 7.43 mm NaH_2_PO_4_, 62 mm NaCl, 1 mm EDTA, and 1 mm dithiothreitol) and sonicated at 40 W and 70% duty cycle for about 2 min, and then clarified by centrifugation at 10 000 *g* for 15 min. Measurements were performed by HPLC analysis using Waters AccQ.Fluor derivatizing reagents (Waters Corp., Milford, MA, USA). The limit of detection is 0.02 nmol, and linearity extends to 5000 nmol. All of the samples were analyzed in triplicate, and the amount of the analyte in the samples normalized by mg protein. Precolumn derivatization of samples and HPLC analysis: an internal standard (10 μL of 2 μm 1,7‐diaminoheptane) was added to 10–60 μL of mixtures of de‐proteinized biological extracts; and borate buffer (0.2 m sodium borate, 1 mm EDTA, pH 8.8) was added to produce final volumes of 80 μL. The AccQ‐Fluor reagent (20 μL) was added and the sample mixed by vortexing and analyzed within 24 h. The HPLC analysis and gradient were obtained as previously described (Skelly *et al*., 2003; Moncada *et al*., 2008).

LTB4 levels were assayed by a specific and sensitive enzyme‐linked immunosorbent assay (ELISA) kit (Assay Designs Inc., Ann Arbor, MI, USA), following the manufacturer's instruction and as previously described (Chu & Praticò, 2011). Aβ1–40 and Aβ1–42 levels were assayed by a sensitive sandwich ELISA kits (WAKO Chem., Richmond, VA, USA), as previously described (Chu *et al*., 2012a). Analyses were performed in duplicate and in a coded fashion.

### 5LO mRNA and Quantitative Real‐time RT–PCR

RNA was extracted and purified using the RNeasy mini‐kit (Qiagen, Valencia, CA, USA), as previously described (Chu *et al*., 2012a,b). Briefly, 1 μg of total RNA was used to synthesize cDNA in a 20 μL reaction using the RT2 First Strand Kit for RT–PCR (SuperArray Bioscience, Frederick, MD, USA). 5‐Lipoxygenase genes were amplified using the proper primers obtained from SuperArray Bioscience. β‐Actin was used as an internal control gene to normalize for the amount of RNA. Quantitative real‐time RT–PCR (qRT–PCR) was performed using StepOnePlus Real‐Time PCR Systems (Applied Biosystems, Foster City, CA, USA). Two microliters of cDNA was added to 10 μL of SYBR Green PCR Master Mix (Applied Biosystems). Each sample was run in duplicate, and analysis of relative gene expression was carried out by StepOne software v2.1 (Applied Biosystems, Foster City, CA, USA).

### 5LO DNA Methylation assay

To measure the 5‐LO DNA methylation levels, we used the restriction digest‐qPCR assay as previously described (Dzitoyeva *et al*., 2009). Briefly, the method utilizes the ability of methylation‐sensitive endonucleases to digest only un‐methylated recognition sites and their inability to act on sites with methylated cytosine. Thus, if the targeted ∗CpG is methylated, the site is blocked for the enzyme's endonuclease activity, and as a result, greater amounts of templates are available for the action of the Taq DNA polymerase. The gene of the mouse 5‐LO promoter to the 5‐untranslated region (UTR) contains the highest C and G densities which have respective recognition sequences cutting sites of methylation‐sensitive endonucleases, such as BstUI (CG ↓ CG) and HpaII (C ↓ CGG) (their respective recognition sequences cutting sites are shown in parentheses). The assay was performed as follows. Genomic DNA was extracted from the mouse brain cortex or N2A‐APPswe cells using PureLink Genomic DNA Mini kit (Invitrogen) according to the manufacturer's protocol. One microgram of genomic DNA was used in a restriction digest reaction for two methylation‐sensitive endonucleases, *Bst*UI, and *Hpa*II. Digested DNA samples were diluted with water, and an aliquot (100 ng DNA) was used for qPCR with SYBR Green PCR Master Mix (Applied Biosystems) as described in the method of quantitative real‐time RT–PCR.

### Data analysis

Data analyses were performed using SigmaStat for Windows version 3.00 (La Jolla, CA, USA). Statistical comparisons were performed by unpaired Student's *t*‐test or the Mann–Whitney rank sum test when a normal distribution could not be assumed. Values represent mean ± SEM. Significance was set at *P* < 0.05.

## Author contributions

J.‐G.L. and D.P. designed the studies; J.‐G.L. executed most of the experiments; C.B and S.M. were involved in the measurement of SAM and SAH; S.G. and W.D.K. provided the cbs/‐mice; J.‐G.L. and D.P. wrote the manuscript. All the authors have seen and approved the final version of the manuscript.

## Conflict of interest

None declared.

## Funding

This work was in part supported by grants from National Institute of Health, HL112966 and AG 51684 (DP), CA06927, and GM098772, and an appropriation from the Commonwealth of Pennsylvania (WDK).

## References

[acel12550-bib-0001] Alzheimer's Association (2015) Alzheimer's diseases facts and figures. Alzheimer's Dement. 2015, 332–384. 10.1016/j.jalz.2015.02.003 25984581

[acel12550-bib-0002] Beydoun MA , Beydoun HA , Gamaldo AA , Teel A , Zonderman AB , Wang Y (2014) Epidemiologic studies of modifiable factors associated with cognition and dementia: systematic review and meta‐analysis. BMC Public Health 14, 643. 24962204 10.1186/1471-2458-14-643 PMC4099157

[acel12550-bib-0003] Boldyrev AA , Johnson P (2007) Homocysteine and its derivatives as possible modulators of neuronal and non‐neuronal cell glutamate receptors in Alzheimer's disease. J. Alzheimers Dis. 11, 219–228. 17522446 10.3233/jad-2007-11209

[acel12550-bib-0004] Castro R , Rivera I , Martins C , Struys EA , Jansen EE , Clode N , Graça LM , Blom HJ , Jakobs C , de Almeida IT (2005) Intracellular S‐adenosylhomocysteine increased levels are associated with DNA hypomethylation in HUVEC. J. Mol. Med. 83, 831–836. 15976919 10.1007/s00109-005-0679-8

[acel12550-bib-0005] Chinnici C , Yao Y , Praticò D (2007) The 5‐lipoxygenase enzymatic pathways in the mouse brain: young versus old. Neurobiol. Aging 28, 1457–1462. 16930777 10.1016/j.neurobiolaging.2006.06.007

[acel12550-bib-0006] Chu J , Praticò D (2011) Pharmacologic blockade of 5‐lipoxygenase improves the amyloidotic phenotype of an Alzheimer's disease transgenic mouse model, involvement of γ‐secretase. Am. J. Pathol. 178, 1762–1769. 21435457 10.1016/j.ajpath.2010.12.032 PMC3078454

[acel12550-bib-0007] Chu J , Giannopoulos PF , Ceballos‐Diaz C , Golde TE , Praticò D (2012a) 5‐Lipoxygenase gene transfer worsens memory, amyloid and tau brain pathologies in a mouse model of Alzheimer disease. Ann. Neurol. 72, 442–454. 23034916 10.1002/ana.23642 PMC3464917

[acel12550-bib-0008] Chu J , Zhuo JM , Pratico D (2012b) Transcriptional regulation of β secretase‐1 by 12/15‐lipoxygenase results in enhanced amyloidogenesis and cognitive impairments. Ann. Neurol. 71, 57–67. 22275252 10.1002/ana.22625 PMC3270901

[acel12550-bib-0009] Chu J , Li J‐G , Praticò D (2013) Zileuton improves memory deficits, amyloid and tau pathology in a mouse model of Alzheimer's disease with plaques and tangles. PLoS One 8, e70991. 23951061 10.1371/journal.pone.0070991 PMC3737232

[acel12550-bib-0010] Chu J , Li J‐G , Joshi YB , Giannopoulos PF , Hoffman NE , Madesh M , Praticò D (2015) Gamma secretase‐activating protein is a substrate for caspase‐3: implications for Alzheimer's disease. Biol. Psychiatry 77, 720–728. 25052851 10.1016/j.biopsych.2014.06.003 PMC4268092

[acel12550-bib-0011] Clarke R , Bennett D , Parish S , Lewington S , Skeaff M , Eussen SJ , Lewerin C , Stott DJ , Armitage J , Hankey GJ , Lonn E , Spence JD , Galan P , de Groot LC , Halsey J , Dangour AD , Collins R , Grodstein F (2014) Effects of homocysteine lowering with B vitamins on cognitive aging: meta‐analysis of 11 trials with cognitive data on 22,000 individuals. Am. J. Clin. Nutr. 100, 657–666. 24965307 10.3945/ajcn.113.076349 PMC4095663

[acel12550-bib-0012] Caudill MA , Wang JC , Melnyk S , Pogribny IP , Jernigan S , Collins MD , Santos‐Guzman J , Swendseid ME , Cogger EA , James SJ (2001) Intracellular S‐adenosylhomocysteine concentrations predict global DNA hypomethylation in tissues of methyl‐deficient cystathionine beta‐synthase heterozygous mice. J. Nutr. 131, 2811–2818. 11694601 10.1093/jn/131.11.2811

[acel12550-bib-0013] Di Meco A , Lauretti E , Vagnozzi A , Praticò D (2014) Zileuton restores memory impairments and reverses amyloid and tau pathology in aged AD mice. Neurobiol. Aging 35, 2458–2464. 24973121 10.1016/j.neurobiolaging.2014.05.016 PMC4171192

[acel12550-bib-0014] Dzitoyeva S , Imbesi M , Ng LW , Manev H (2009) 5‐Lipoxygenase DNA methylation and mRNA content in the brain and heart of young and old mice. Neural. Plast. 2009, 209596. 20052386 10.1155/2009/209596 PMC2801004

[acel12550-bib-0015] Fuso A , Seminara L , Cavallaro RA , D'Anselmi F , Scarpa S (2005) S‐adenosylmethionine/homocysteine cycle alterations modify DNA methylation status with consequent deregulation of PS1 and BACE and beta‐amyloid production. Mol. Cell. Neurosci. 28, 195–204. 15607954 10.1016/j.mcn.2004.09.007

[acel12550-bib-0016] Giannopoulos PG , Praticò D (2015) Alzheimer's disease In Diet Nutrition and Dementia (Martin CR, Reddy V, eds). London, UK: Elsevier Publisher, pp. 13–21.

[acel12550-bib-0017] Giannopoulos PF , Chu J , Joshi YB , Sperow M , Li JG , Kirby LG , Praticò D (2014) Gene knockout of 5‐lipoxygenase rescues synaptic dysfunction and improves memory in the triple‐transgenic model of Alzheimer's disease. Mol. Psych. 19, 511–518. 10.1038/mp.2013.23 PMC3688674 23478745

[acel12550-bib-0018] Kim HJ , Cho HK , Kwon YH (2008) Synergistic induction of ER stress by homocysteine and beta‐amyloid in SH‐SY5Y cells. J. Nutr. Biochem. 19, 754–761. 18430556 10.1016/j.jnutbio.2007.09.009

[acel12550-bib-0019] Kuszczyk M , Gordon‐Krajcer W , Lazarewicz JW (2009) Homocysteine‐induced acute excitotoxicity in cerebellar granule cells in vitro is accompanied by PP2A‐mediated de‐phosphorylation of tau. Neurochem. Int. 55, 174–180. 19428823 10.1016/j.neuint.2009.02.010

[acel12550-bib-0020] Li J‐G , Chu J , Barrero C , Merali S , Praticò D (2014) Homocysteine exacerbates Aβ, tau pathology and cognitive deficit in a mouse model of Alzheimer's with plaques and tangles. Ann. Neurol. 75, 851–863. 24644038 10.1002/ana.24145 PMC4362695

[acel12550-bib-0021] Moncada CA , Clarkson A , Perez‐Leal O , Merali SJ (2008) Mechanism and tissue specificity of nicotine‐mediated lung D‐adenosylmethionine reduction. Biol. Chem. 283, 7690–7696. 10.1074/jbc.M709399200 18180293

[acel12550-bib-0022] Oddo S , Caccamo A , Shepherd JD , Murphy MP , Golde T , Kayed R , Metherate R , Mattson M , Akbari Y , LaFerla F (2003) Triple transgenic model of Alzheimer's disease with plaques and tangles: intracellular Abeta and synaptic dysfunction. Neuron 39, 409–421. 12895417 10.1016/s0896-6273(03)00434-3

[acel12550-bib-0023] Parnetti L , Bottiglieri T , Lowenthal D (1997) Role of homocysteine in age‐related vascular and non‐vascular diseases. Aging Clin. Exp. Res. 9, 241–257. 10.1007/BF03341827 9359935

[acel12550-bib-0024] Perna AF , *et al* (2003) Homocysteine and oxidative stress. Amino Acids 25, 409–417. 14661100 10.1007/s00726-003-0026-8

[acel12550-bib-0025] Qu T , Manev R , Manev H (2001) 5‐Lipoxygenase (5‐LOX) promoter polymorphism in patients with early‐onset and late‐onset Alzheimer's disease. J. Neuropsychiatry Clin. Neurosci. 13, 304–305. 11449041 10.1176/jnp.13.2.304

[acel12550-bib-0026] Ravaglia G , Forti P , Maioli F , Muscari A , Sacchetti L , Arnone G , Nativio V , Talerico T , Mariani E (2003) Homocysteine and cognitive function in healthy elderly dwellers in Italy. Am. J. Clin. Nutr. 77, 668–673. 12600859 10.1093/ajcn/77.3.668

[acel12550-bib-0027] Ravaglia G , Forti P , Maioli F , Servadei L , Martelli M , Arnone G , Talerico T , Zoli M , Mariani E (2004) Plasma homocysteine and inflammation in elderly patients with cardiovascular disease and dementia. Exp. Geront. 39, 443–450. 10.1016/j.exger.2003.11.005 15036404

[acel12550-bib-0028] Gupta S , Kühnisch J , Mustafa A , Lhotak S , Schlachterman A , Slifker MJ , Klein‐Szanto A , High KA , Austin RC , Kruger WD (2009) Mouse models of cystathionine β‐synthase deficiency reveal significant threshold effects of hyperhomocysteinemia. FASEB J. 23, 883–989. 18987302 10.1096/fj.08-120584 PMC2653989

[acel12550-bib-0029] Selhub J (1999) Homocysteine metabolism. Ann. Rev. Nutr. 19, 217–246. 10448523 10.1146/annurev.nutr.19.1.217

[acel12550-bib-0030] Shen L , Ji HF (2015) Association between homocysteine, folic acid, vitamin B12 and Alzheimer's disease: insights from meta‐analysis. J. Alzheimers Dis. 46, 777–790. 25854931 10.3233/JAD-150140

[acel12550-bib-0031] Skelly M , Hoffman J , Fabbri M , Holzman RS , Clarkson AB , Merali S (2003) S‐adenosylmethionine concentrations in diagnosis of *Pneumocystis carini* pneumonia. Lancet 361, 1267–1268. 12699956 10.1016/S0140-6736(03)12984-4

[acel12550-bib-0032] Turner MA , Yang X , Yin D , Kuczera K , Borchardt RT , Howell PL (2000) Structure and function of S‐adenosylhomocysteine hydrolase. Cell Biochem. Biophys. 33, 101–125. 11325033 10.1385/CBB:33:2:101

[acel12550-bib-0033] Uhl J , Klan N , Rose M , Entian KD , Werz O , Steinhilber D (2002) The 5‐lipoxygenase promoter is regulated by DNA methylation. J. Biol. Chem. 277, 4374–4379. 11706027 10.1074/jbc.M107665200

[acel12550-bib-0034] Wang Y , Kavran JM , Chen Z , Karukurichi KR , Leahy DJ , Cole PA (2014) Regulation of S‐adenosylhomocysteine hydrolase by lysine acetylation. J. Biol. Chem. 289, 31361–31372. 25248746 10.1074/jbc.M114.597153 PMC4223336

[acel12550-bib-0035] Zhuo JM , Portugal GS , Kruger WD , Wang H , Gould TJ , Pratico D (2010) Diet‐induced hyperhomocysteinemia increases amyloid‐beta formation and deposition in a mouse model of Alzheimer's disease. Curr. Alzheimer Res. 7, 140–149. 19939226 10.2174/156720510790691326 PMC3880573

[acel12550-bib-0036] Zhuo J , Wang H , Praticò D (2011) Is hyperhomocysteinemia an AD risk factor, an AD marker or neither? Trends Pharmacol. Sci. 32, 562–571. 21684021 10.1016/j.tips.2011.05.003 PMC3159702

